# Determining the functional relevance of *PSEN2* 3′UTR isoforms in Alzheimer's disease

**DOI:** 10.1002/alz70855_105273

**Published:** 2025-12-24

**Authors:** Julianna N Brutman, Marissa J de Leon, Samuel Smukowski, Tina Busald, Eli J Kaufman, Evangelos Nizamis, Paul N Valdmanis

**Affiliations:** ^1^ University of Washington, Seattle, WA, USA

## Abstract

**Background:**

Pathogenic variants in presenilin 1 (*PSEN1*) or presenilin 2 (*PSEN2*) cause familial Alzheimer's disease (AD). Recent long‐read RNA sequencing of *PSEN2* revealed cryptic exon inclusion and differential regulation of the 3′ untranslated region (3′UTR) in sporadic AD. Notably, two‐thirds of *PSEN2* reads harbored the canonical short 3′UTR, while the other third contained an extended ∼4kb long 3′UTR. Given that 3′UTRs contain critical regulatory elements, we sought to determine the functional significance of *PSEN2* 3′UTR isoforms in AD.

**Method:**

To develop *in vitro* models to assess *PSEN2* 3′UTR isoforms, we designed constructs containing the *PSEN2* 3′UTR short and long isoforms to overexpress in either HMC3 human microglial or SH‐SY5Y human neuroblastoma cell lines. Since miRNAs are known to target 3′UTRs, we also completed homogenate and synaptosome small RNA sequencing on AD and control samples to profile miRNA expression. Finally, we tested whether *PSEN2* 3′UTR length or inclusion of cryptic exon 9B altered cellular localization in human frontal cortex samples using BaseScope in‐situ hybridization.

**Result:**

We primarily detected short *PSEN2* in the cytoplasm with significantly reduced levels in AD versus control frontal cortex. Conversely, long *PSEN2* was retained in the nucleus and expressed at similar levels between cases and controls. These findings are relevant for *PSEN2* expression studies as *in vitro* findings indicated increased long *PSEN2* 3′UTR isoform abundance relative to the short *PSEN2* 3′UTR. Small RNA sequencing analysis identified 53 homogenate and 76 synaptosome miRNAs with significant differential regulation in AD, including one miRNA, miR‐34c, that was significantly downregulated in both fractions. The long *PSEN2* 3′UTR contains 10 binding sites for 5 miRNAs with significant differential regulation in AD brains relative to controls, including 3 miRNAs with significant synaptosome downregulation (miR‐346, miR‐326, and miR‐548p), and 2 miRNAs with significant homogenate downregulation (miR‐217) and upregulation (miR‐890).

**Conclusion:**

Collectively, our data establish 1) an *in vitro* model to test *PSEN2* 3′UTR isoforms, 2) miRNA expression differences in human AD synaptosome and homogenate fractions, and 3) decreased *PSEN2* transcript signal in human AD frontal cortex tissue. Elucidating the functional significance of *PSEN2* 3′UTR isoform regulation will further our understanding of AD.

